# Supplementary material for: Expression of the Heterotrimeric GP2/GP3/GP4 Spike of an Arterivirus in Mammalian Cells
Source: Viruses. 2022 Apr 1;14(4):749. doi: 10.3390/v14040749 (PMC9030998; doi:10.3390/v14040749)
Supplement: Supplementary file 1 [file viruses-14-00749-s001.zip › viruses-1583782-supplementary-revised/Figure S2.pdf]

Manders' overlap coefficent

Pearson's correlation coeficient

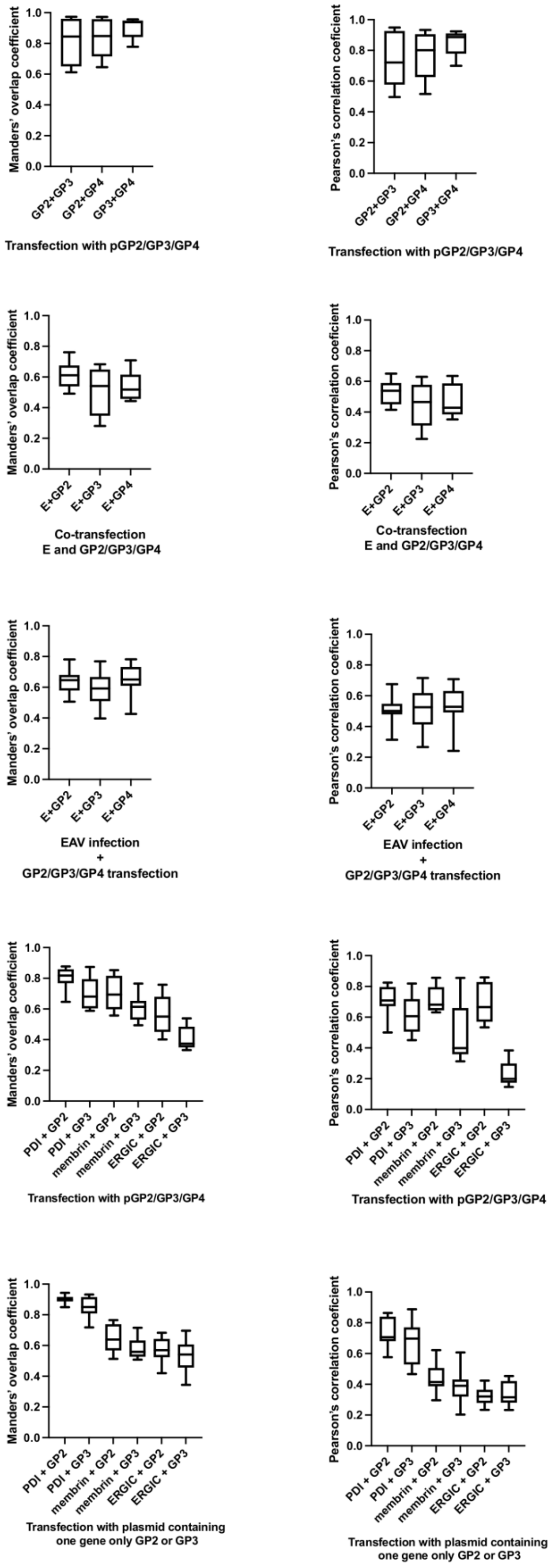

Figure S2: Comparison of the co-localization graphs of Manders' overlap coefficients and Pearson's correlation coefficients.
