# Supplementary material for: Expression of the Heterotrimeric GP2/GP3/GP4 Spike of an Arterivirus in Mammalian Cells
Source: Viruses. 2022 Apr 1;14(4):749. doi: 10.3390/v14040749 (PMC9030998; doi:10.3390/v14040749)
Supplement: Supplementary file 1 [file viruses-14-00749-s001.zip › viruses-1583782-supplementary-revised/Supplementary Table S1 MultiMam R1.pdf]

Table S1: Oligonucleotides used for this study.

Primer name	Sequence 5' > 3 '	Purpose
EML FOR	GCTTACACGCGTATGGGCTTAGTGTGGTC	Primer for cloning E gene into pML-DAZ2
EML REV	GTAAGCTCTAGATTAAACAGGTCTGTAACG	Primer for cloning E gene into pML-DAZ2
EXhoEcoFor	GATCCTCGAGGAATTCATGGGCTTAGTGTGG	Primer for cloning E into pMDK
EMluKpnRev	GATCGGTACCACGCGTTTAAACAGGTCTGTAAC	Primer for cloning E into pMDK
GP3HAXhoBamFor	GCTTACGGATCCCTCGAGATGGGTCGTG	Primer for cloning into pMDS
GP3HAXbaKpnRev	GATCGGTACCTCTAGATTAAGCGTAGTC	Primer for cloning into pMDS
GP2XhoBamFor	GATCGGATCCCTCGAGACGCGTATGC	Cloning GP2-myc into pMDC and pMDK
GP2SmaXbaRev	GCTTACTCTAGACCCGGGCTACAGATCTTCTTCAGAAATAAGTTTTTGCTCCAAAATCTTGCGC	Addition of the tag and cloning of GP2-myc into pMDC
GP2mycMluKpnRev	GATCGGTACCACGCGTCTACAGATCTTCTTCAG	Primer for subcloning GP2-myc into pMDK
GP4XhoMluFor	GCTTACCTCGAGACGCGTATGAAGATCTACGGCTGC	Primer for cloning GP4 into pACEMam2
Gp4KpnSmaRev	GCTTACGGTACCCCCGGGCTACTTGTCATCGTCGTCCTTGTAGTCTAGATAACATCGTTGAGC	Primer for addition of FLAG tag and cloning into pACEMam2
GP4KpnHindRev	GATCTAAAGCTTGGTACCCTATTACTTGTCATCGTCGTCCTTGTAGTCGGTGGCGACCGGTGGATCC	Primer for adding linker to FLAG tag GP4 and cloning into pACEMam2
GP4linkerV5Rev	GTAAGCAAGCTTGGTACCTTACGTAGAATCG AGACCGAGGAGAGGGTTAGGGAT AGGCTTACCGGTGGCGACCGGTGGAT	Primer for exchanging tag to V5 in GP4 and cloning into pACEMam2
