# Supplementary figures and images for: Expression of the Heterotrimeric GP2/GP3/GP4 Spike of an Arterivirus in Mammalian Cells
Source: Viruses. 2022 Apr 1;14(4):749. doi: 10.3390/v14040749 (PMC9030998; doi:10.3390/v14040749)

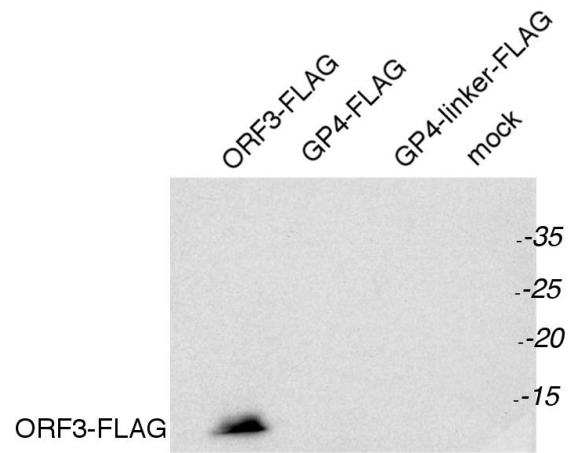

**Figure S1:** Expression of the GP4 with FLAG tag.

Supplement: Supplementary file 1 [file viruses-14-00749-s001.zip › viruses-1583782-supplementary-revised/Figure S1.pdf]

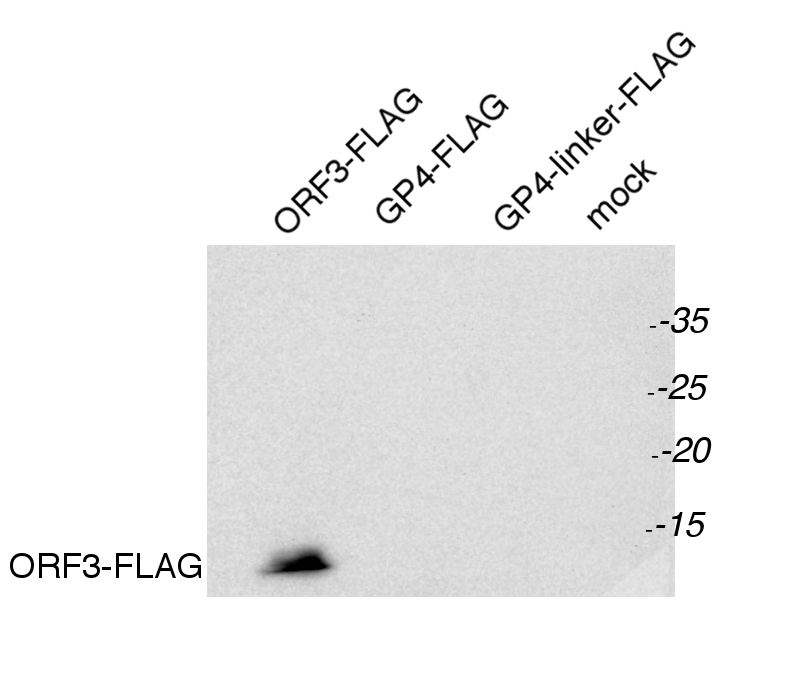

Supplement: Supplementary file 1 [file viruses-14-00749-s001.zip › viruses-1583782-supplementary-revised/Figure S1.tif]

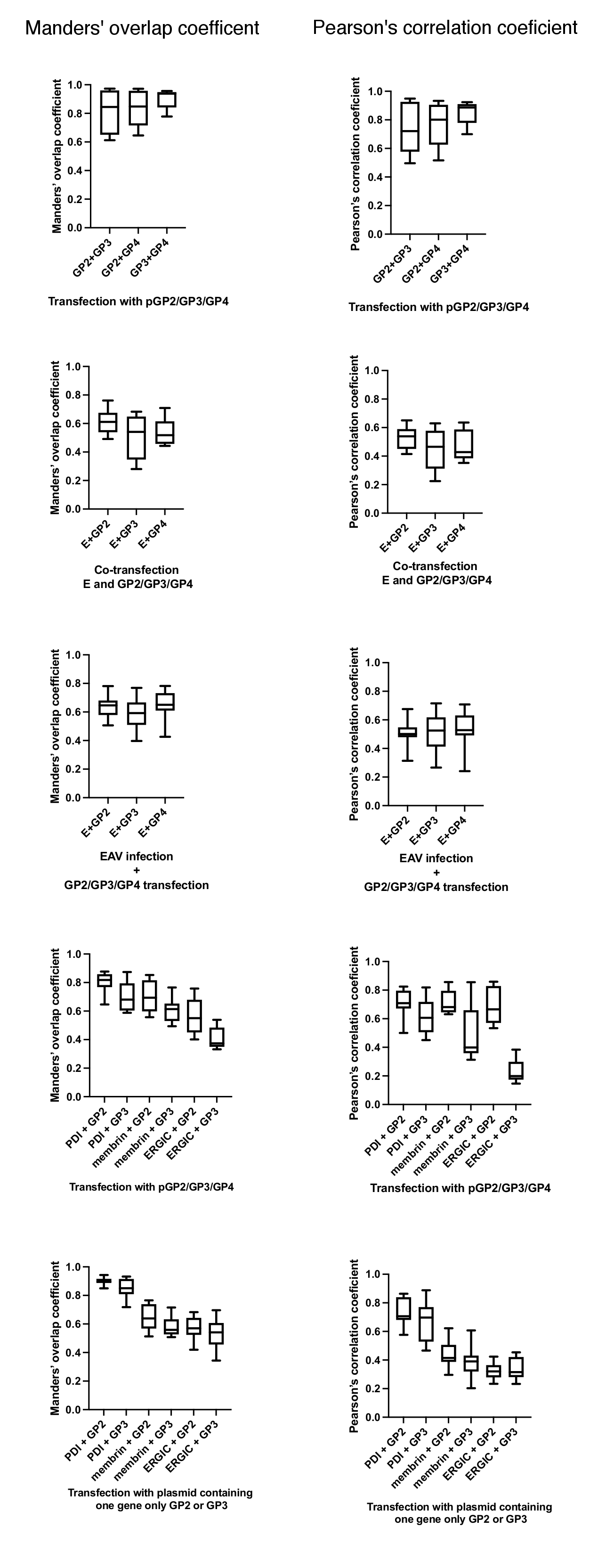

Supplement: Supplementary file 1 [file viruses-14-00749-s001.zip › viruses-1583782-supplementary-revised/Figure S2.tiff]
